# Asymmetric biphasic pulsed field ablation of paroxysmal atrial fibrillation: clinical assessment

**DOI:** 10.3389/fcvm.2026.1945178

**Published:** 2026-10-16

**Authors:** Xinyu Xu, Yunfan Li, Fengmin Lu, Huanming Li, Heng Cai

**Affiliations:** 1 School of Biomedical Engineering and Technology, Tianjin Medical University, Tianjin, China; 2 Department of Cardiology, Tianjin Chest Hospital, Tianjin, China; 3 Department of Cardiology, Tianjin 4th Central Hospital, Tianjin, China; 4 Department of Cardiology, Tianjin Medical University General Hospital, Tianjin, China

**Keywords:** atrial fibrillation, catheter ablation, electroporation, pulmonary vein isolation, pulsed field ablation

## Abstract

**Background:**

Pulsed field ablation (PFA) is a non-thermal, tissue-selective therapy for atrial fibrillation. In this study, we evaluated the safety and efficacy of a novel asymmetric biphasic PFA system, which combines a unique pulse-parameter configuration with a dedicated, adjustable six-spline catheter.

**Methods:**

This prospective, multicenter, single-arm study was conducted in China. Patients with drug-refractory paroxysmal AF underwent pulmonary vein isolation (PVI) using a 1,000 V asymmetric biphasic PFA system with a novel parameter configuration. The primary effectiveness endpoint was freedom from atrial arrhythmia recurrence through 12 months post-blanking. The primary safety endpoint was predefined serious adverse events. The secondary safety endpoint was hemolysis and silent cerebral lesions (SCLs).

**Results:**

Among 120 enrolled patients, 105 underwent ablation and completed the 12-month follow-up, while 15 patients withdrew before the PFA operation. Acute PVI was successfully achieved in all 420 targeted veins. At 12 months, the Kaplan–Meier estimate of freedom from atrial arrhythmia recurrence was 69.5% (95% CI, 60.1–77.5%). The observed 12-month effectiveness was within the range reported in previous studies of PFA technologies. Regarding safety, major procedural complications—including pulmonary vein stenosis, phrenic nerve injury, atrioesophageal fistula, stroke, and acute kidney injury (AKI)—were entirely absent. However, one patient (0.95%) experienced a transient coronary vasospasm that was successfully resolved with an intracoronary vasodilator. Additionally, SCLs were detected in two patients (1.9%). While laboratory hemolysis was a common finding, it did not lead to renal impairment.

**Conclusions:**

This asymmetric biphasic PFA system achieved complete acute PVI and 12-month effectiveness was within the range reported in previous studies of PFA technologies. It showed a favorable safety profile, with few SCLs and no hemolysis-related AKI.

**Clinical Trial Registration:**

https://www.chictr.org.cn/showproj.html?proj=134973, identifier ChiCTR2200062157.

## Introduction

Pulsed field ablation (PFA) is a non-thermal, tissue-selective cardiac ablation modality based on the principles of irreversible electroporation. Because distinct tissue types possess unique electrical threshold field strengths to induce necrosis, PFA exhibits inherent tissue preferentiality. Preclinical and clinical studies have demonstrated the feasibility of PFA for durable pulmonary vein isolation (PVI), highlighting a favorable safety profile that minimizes collateral damage to adjacent structures like the esophagus, phrenic nerve, and coronary arteries (1–7).

Early PFA systems relied on monophasic pulses for their simple design and high electroporation efficiency. However, these systems caused significant skeletal muscle contraction, nerve stimulation, and adverse electrochemical reactions like electrode polarization and gas formation (8). To address these limitations, biphasic waveforms—featuring two phases but opposite polarity—were developed. By balancing electrical charge, this design minimizes electrochemical reactions and muscle stimulation, significantly improving safety and patient tolerability (9). Consequently, biphasic pulses dominate contemporary clinical platforms, including FARAPULSE (Boston Scientific), VARIPULSE (Biosense Webster), and PulseSelect (Medtronic) (10).

While commercial biphasic platforms have proven highly effective, standard symmetric biphasic configurations inherently introduce unique biophysical trade-offs. In generic symmetric designs, the delivery of equal and opposite electrical phases leads to several documented limitations: (1) The charge cancellation effect. The phenomenon where the second opposite-phase pulse neutralizes the transmembrane potential accumulation from the first phase is extensively documented (11, 12). (2) Elevated lethal electric field thresholds. Because of the cancellation effect, symmetric biphasic pulses require significantly higher electric field strengths (V/cm) to achieve irreversible cell death compared to monophasic pulses (8). (3) Unexpected thermal profile and Joule heating risks. While PFA is fundamentally marketed as a non-thermal modality, compensating for biphasic inefficiencies by packing a high density of high-frequency pulse bursts triggers unintentional Joule heating. A clinical review highlights that heavy energy delivery profiles can cause unexpected thermal effects, microbubble formation (increasing silent cerebral embolism risks), and acute hemolysis (13).

To overcome these limitations of standard symmetric waveforms, we developed a novel asymmetric biphasic PFA system integrating a re-engineered pulse-parameter configuration and a dedicated, adjustable six-spline catheter. Having previously validated the efficacy and safety of this asymmetric platform *in vitro* and *in vivo* (14, 15), this multi-center clinical trial aims to evaluate its safety and clinical efficacy in human subject.

## Methods

### Ablation system

The PFA system consists of a pulse generator, a 10.5 F PFA catheter, and a 12F steerable sheath (Tianjin Intelligent Health Co., Ltd., Tianjin, China). The asymmetric biphasic PFA system applied an optimized pulse waveform. Each pulse consisted of a microsecond-scale biphasic waveform, comprising a 7 *μ*s positive phase and a 3 μs negative phase, with a 5 μs interval between the two phases. The pulse amplitude was 1,000 V (Figure 1). The PFA catheter is designed with six splines, each incorporating a single electrode on the outer surface (six electrodes in total) to ensure optimal tissue apposition and maximize ablation efficacy (Figure 2). Pulsed field energy is delivered through all electrodes. During each pulse, three electrodes serve as positive electrodes and three as negative electrodes. The catheter can be partially expanded and then advanced into the pulmonary vein for ablation. Its diameter is adjusted to fit the vein for optimal tissue apposition (Figure 3A). When fully deployed in the flower configuration, the catheter reaches a maximum diameter of 32 mm. Then the catheter can be positioned at the ostium for ablation (Figure 3B). The catheter is advanced through a guidewire to maximize tissue contact.

**Figure 1:**
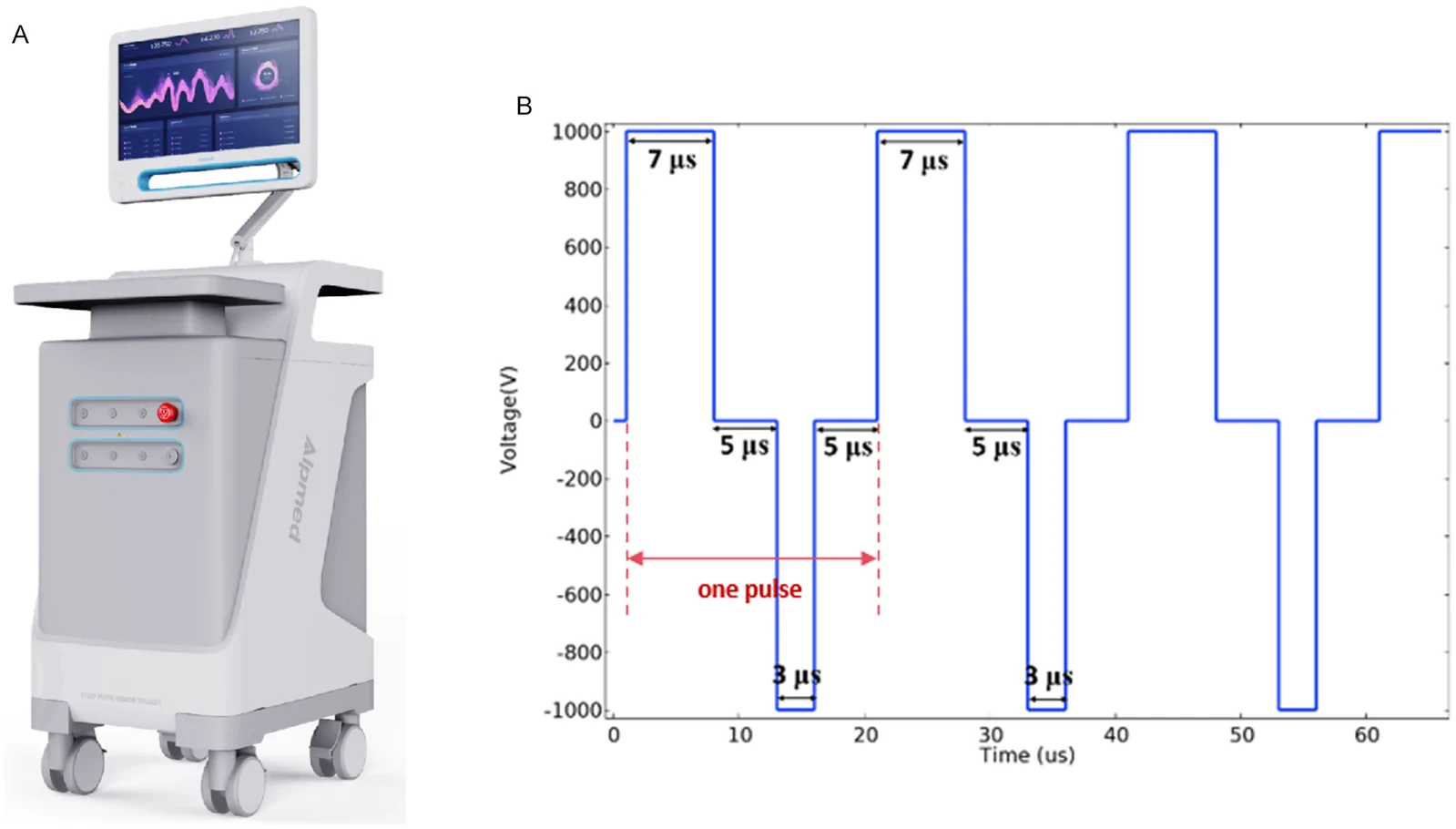
The PFA ablation system. **(A)** The pulse generator. **(B)** The asymmetric biphasic waveform comprises a 7 μs positive pulse and a 3 μs negative pulse, with a 5 μs interval between the two phases.

**Figure 2:**
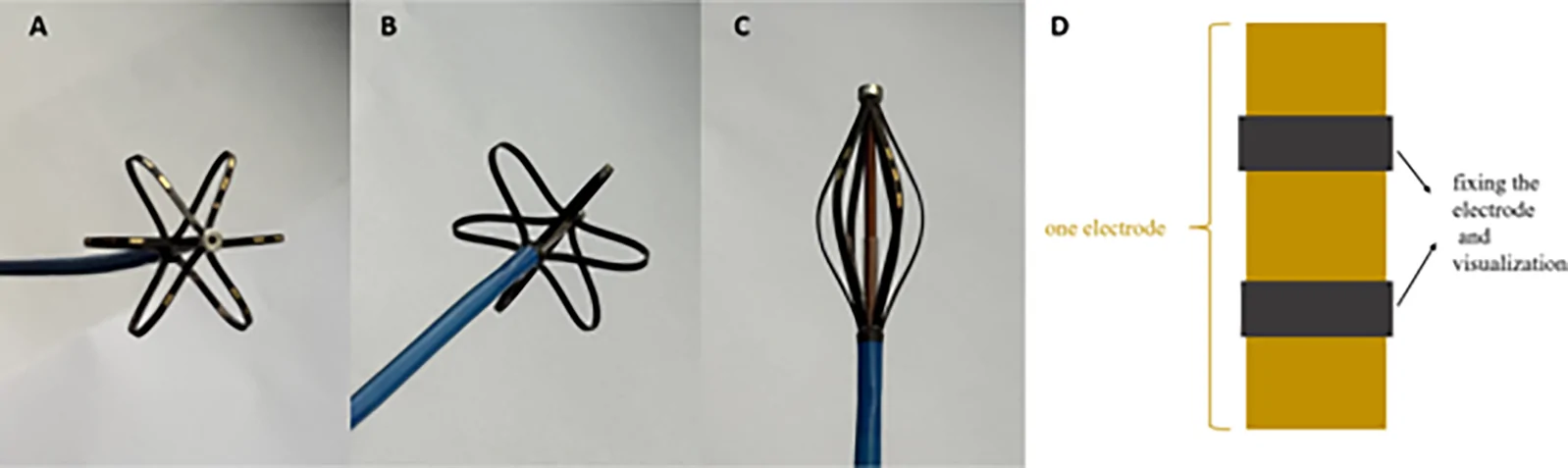
The PFA catheter. **(A)** Fully deployed 6-spline PFA catheter in flower configuration. **(B)** Fully deployed in the flower configuration, as viewed from the distal end. **(C)** The 6-spline PFA catheter is half deployed in American football configuration. **(D)** Schematic illustration of the electrode structure.

**Figure 3:**
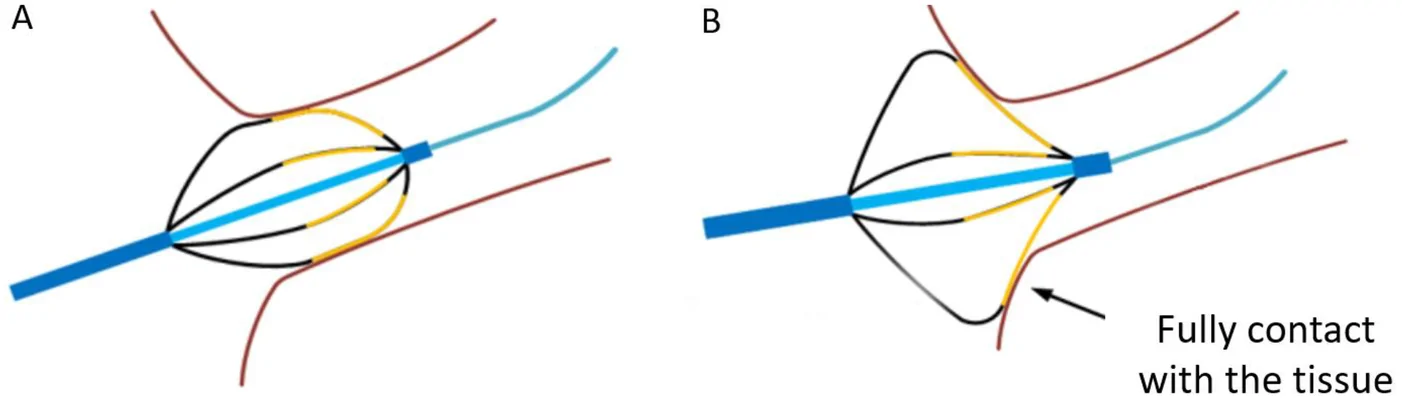
Schemas of catheter ablation. **(A)** Pulmonary vein myocardial sleeve. **(B)** Pulmonary vein ostium.

### Trial design

The clinical trial (ChiCTR2200062157) was a prospective, multicenter, single-arm study conducted in China. It was designed to determine the safety and efficacy of asymmetric biphasic waveform PFA system in Chinese patients with a documented history of paroxysmal AF. Patients were enrolled between January 2022 and December 2024 at 10 participating centers across China. The number of patients enrolled at each center ranged from 1 to 24, with a total of 120 patients enrolled (see Table 1). Before the ablation procedure, 15 patients withdrew due to voluntary withdrawal or the identification of other medical conditions that precluded continuation. Therefore, 105 patients underwent ablation and completed the prespecified 12-month follow-up and were included in the final analysis. All procedures were performed by experienced electrophysiologists who had prior experience with PFA. A flow diagram illustrating patient enrollment and the final analysis population is provided in Figure 4.

**Table 1:** Participating centers and patient enrollment.

Center	Enrolled, *n*	Treated, *n*
Tianjin First Central Hospital	15	13
Tianjin Medical University General Hospital	15	15
Tianjin Medical University Second Hospital	1	1
Taiyuan Central Hospital	3	2
Luoyang Central Hospital	1	1
Cangzhou Central Hospital	17	14
The First Affiliated Hospital of Harbin Medical University	10	8
Henan University Huaihe Hospital	18	15
Tianjin Chest Hospital	31	29
Tianjin Fourth Central Hospital	9	7

**Figure 4:**
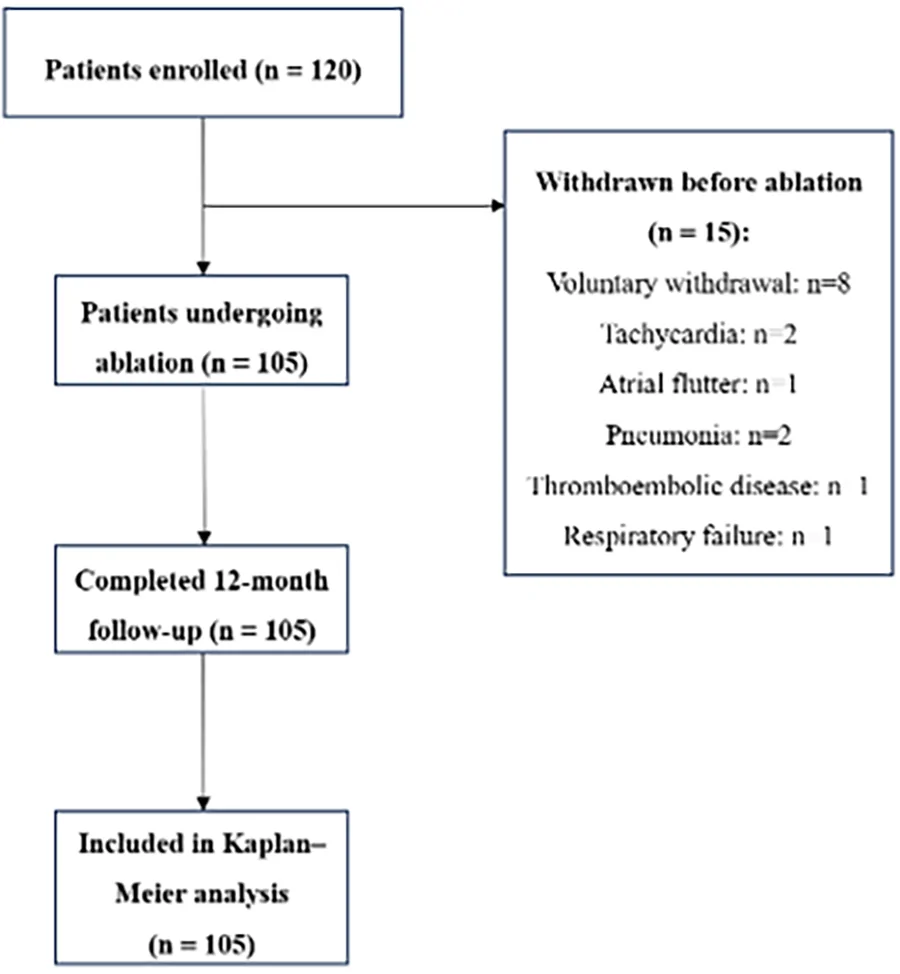
Flowchart of patient enrollment, ablation, and 12-month follow-up.

The clinical protocol was approved by the ethics committee at each clinical trial site and national regulatory agencies. Eligible patients had symptoms of paroxysmal AF and were refractory or intolerant to at least one class I or III antiarrhythmic drugs (AADs). The full list of inclusion and exclusion criteria is given in Table 2.

**Table 2:** Inclusion and exclusion criteria.

Inclusion criteria	Exclusion criteria
Patients with: Paroxysmal AF: Recurrent AF (≥ 2 episodes) that terminates spontaneously within seven days Patients who have failed or are intolerant to at least one class I or class III AAD Patients who are ≥ 18 and ≤ 80 years of age on the day of enrollment Patient participation requirements: (a) Is willing and capable of providing Informed Consent to undergo study procedures. (b) Is willing to participate in all examinations and follow-up visits and tests associated with this clinical study	Left ventricular ejection fraction ≤ 40% Contraindications to cerebral MRI examination NYHA Class III or IV CHF Previous ablation or surgery for AF Women of childbearing potential who are pregnant, lactating or planning to get pregnant within 1 year Significant bleeding and/or clotting disorder Clinically significant infection, and any other significant uncontrolled or unstable medical condition (such as, hyperthyroidism) Active malignancy or history of treated cancer Estimate glomerular filtration rate (eGFR) < 60 mL/min/1.73 m2 or has ever received dialysis. Clinically significant arrhythmias other than AF Complicated with severe organic cardiovascular disease History of stroke or TIA within prior 3 months Thromboembolic disease Clinically significant psychological condition and/or epilepsy Current or anticipated enrollment in any other clinical study Any other medical conditions that would prevent participation in the study, interfere with assessment or therapy, significantly raise the risk of study participation, or confound data or its interpretation as determined by study investigators

AF, atrial fibrillation; AAD, antiarrhythmic drugs; NYHA, New York Heart Association; CHF, congestive heart failure; TIA, transient ischemic attack.

### Blood sampling and laboratory measurements

Blood samples were collected at baseline and on the first day after the procedure to assess markers of hemolysis and renal function, including lactate dehydrogenase (LDH), haptoglobin (Hp), hemoglobin, total bilirubin, and creatinine. Additionally, free plasma hemoglobin (fHb) was measured using an azide methemoglobin spectrophotometric assay (HemoCue Plasma/Low Hb system) both before and immediately after the procedure.

Hemolysis was defined as a decrease in serum haptoglobin (Hp) level of >10 mg/dL from baseline to postoperative day 1 (16).

### Ablation procedure

After informed consent, all patients underwent preoperative computed tomography (CT) scanning or echocardiography to measure left atrial (LA) size and PV anatomy. Procedures were performed under general anesthesia or conscious sedation. All patients received uninterrupted oral anticoagulation, and intravenous heparin was administered to maintain an activated clotting time ≥300 s throughout the procedure. During the procedure, pre- and post-ablation diaphragm motion was evaluated to observe for changes in phrenic nerve function. After a single transseptal puncture, the 12F deflectable PFA sheath was inserted into the LA. Ablation was performed in a clockwise order: left superior, left inferior, right inferior, and right superior pulmonary vein. For each vein, the catheter was first placed at the myocardial sleeves in the basket configuration. Its diameter was adjusted to fit the pulmonary vein. Then 50 pulses were delivered. The catheter was withdrawn and reshaped into the flower configuration. It was then repositioned at the ostium for delivery of 100 pulses. Positioning was guided by intracardiac echocardiography (ICE) and fluoroscopy. Prophylactic nitroglycerin was not routinely administered. Nitroglycerin was used only as a therapeutic intervention in patients who developed coronary vasospasm. All patients undergoing PFA received standardized intravenous hydration during the peri-procedural period. Normal saline was infused at a rate of 2 mL/kg/h during the procedure and continued for 12–24 h after the procedure. After completion of PVI, a 20-minute waiting period was routinely applied. Pulmonary vein isolation was reassessed by confirming both entrance and exit block at the end of the waiting period. If acute PV reconnection was detected during this period, additional PFA applications were delivered until bidirectional PV isolation was re-established. In selected patients, anatomical voltage maps of the left atrium were created using the CARTO system before and after PFA to verify the ablation effect.

### Follow-up

Postprocedural anticoagulation was managed according to the 2017 HRS/EHRA/ECAS/APHRS/SOLAECE expert consensus recommendations (17). Oral anticoagulation was continued for at least 2 months after ablation, with subsequent continuation determined by individual thromboembolic risk.

All patients underwent brain MRI 12–48 h after ablation to assess for newly developed silent cerebral lesions (SCLs). A preprocedural brain MRI was used to identify pre-existing acute cerebral lesions. Brain MRI was performed using a 3.0-T MRI scanner with a slice thickness of 5 mm. SCLs were defined as newly detected acute cerebral lesions on postprocedural MRI, characterized by focal hyperintensity on diffusion-weighted imaging (DWI) with corresponding reduced apparent diffusion coefficient (ADC) values. Patients with pre-existing acute cerebral lesions identified on preprocedural MRI were excluded from the SCL analysis. Brain MRI was also performed in all patients at 3 and 6 months after ablation to assess for newly detected cerebral lesions and interval changes. All MRI scans were reviewed by experienced radiologists at the participating centers. The image assessors were not specifically blinded to the patients' clinical or procedural information. The occurrence of transient ischemic attack (TIA) was assessed clinically during follow-up.

Postprocedural esophagogastroduodenoscopy (EGD) was performed in all patients on postoperative day 1 to assess acute esophageal injury and repeated at 3 months to evaluate late esophageal changes. Esophageal injury was defined as any endoscopic evidence of esophageal erosion, ulceration, or perforation.

Patients returned for follow-up visits at 3 months, 6 months and 12 months. Follow-up assessments included screening for adverse events, recurrent arrhythmias, medication changes, electrocardiography, new hospital visits. Patients with arrhythmia recurrence within the 90-day blanking period were not considered therapeutic failures, and additional ablation was not performed during this period. Class I or III antiarrhythmic drugs were allowed during the blanking period, but not after 90 days. A repeat CT/MRI was performed at 3 months and 6 months visit to assess for PV stenosis.

Rhythm monitoring was performed using 24-hour Holter monitoring and 12-lead ECG during follow-up. All patients underwent 24-hour Holter monitoring at 6 and 12 months after ablation. In addition, 12-lead ECG was performed every 2–3 weeks from 3 to 6 months and approximately every 2 months from 6 to 12 months after the 90-day blanking period.

### Endpoints

The primary effectiveness endpoint was freedom from atrial arrhythmia recurrence during the follow-up period (post-blanking period to the end of the 12-month follow-up). Freedom from atrial arrhythmia was defined as: (1) freedom from AF/atrial flutter (AFL)/atrial tachycardia (AT) lasting ≥30 s in duration documented by protocol-specified 24-hour Holter monitoring or12-lead ECG; (2) freedom from re-ablation or surgical treatment for AF/AFL/AT; (3) freedom from the use of Class I or III antiarrhythmic drugs during the post-blanking evaluation period.

The secondary effectiveness endpoint was the proportion of subjects that achieve acute PVI. Acute PVI success was defined as maintaining electrical isolation of all PVs 20 min after the ablation procedure.

The primary safety endpoint was the incidence of the predefined serious adverse events. The predefined serious adverse events include death, peripheral or organ thromboembolism, persistent phrenic nerve palsy, stroke, transient ischemic attack, cardiac tamponade or perforation, myocardial infarction, coronary vasospasm, pericarditis, and pulmonary edema occurring within 7 days of the ablation procedure, as well as pulmonary vein stenosis (>70% diameter reduction) and/or atrioesophageal fistula occurring at any time during the 12-month follow-up period.

The secondary safety endpoint was the occurrence of hemolysis and SCLs.

### Statistical analysis

#### Minimum sample size calculation

This prospective, multicenter, single-arm pivotal study used a performance-goal approach for the primary effectiveness endpoint. The performance goal (P₀ = 50%) was based on the 2017 HRS/EHRA/ECAS/APHRS/SOLAECE expert consensus statement (17), which recommended a minimum chronic acceptable success rate of 50% at 12 months for patients with paroxysmal atrial fibrillation when a minimum chronic success rate is used as the objective effectiveness criterion. The expected effectiveness rate (P₁ = 65%) was conservatively selected based on the PULSED AF Pivotal Trial (18), which reported a 1-year effectiveness rate of 66.2% (95% CI, 57.9–73.2%) in patients with paroxysmal atrial fibrillation. The sample size was calculated using a one-sided exact binomial test with a significance level of *α* = 0.05% and 80% power (*β* = 0.20) to demonstrate superiority over the prespecified performance goal of 50%, assuming an expected effectiveness rate of 65%. Under these assumptions, 69 evaluable patients were required. Considering an anticipated 20% attrition rate, the minimum target enrollment was 87 patients. Ultimately, 120 patients were enrolled to provide additional robustness for the primary endpoint analysis.

#### Efficacy and safety analyses

Continuous data are expressed as mean ± SD, median, minimum and maximum values. Categorical data are expressed as a proportion (%). Independent categorical variables were compared using Pearson's chi-square test or Fisher's exact test. Ranked data are expressed as Wilcoxon rank sum test or Cochran-Mantel-Haenszel test. The primary effectiveness outcome was evaluated with Kaplan–Meier analysis. Time 0 was defined as the date of ablation, and data were censored at the patient's 12-month follow-up or exit from the registry. The start date for assessing atrial arrhythmia was 91 days after time 0. All tests of significance were two-tailed. For the secondary laboratory comparisons, statistical significance was defined as *P* ≤ 0.0083 after Bonferroni correction.

## Result

### Patient characteristics

As shown in Table 3, the mean age of the cohort was 63.7 ± 8.33 years, 60% were men, and the mean CHA2DS2-VASC score [congestive heart failure, hypertension, age ≥75 years [doubled], diabetes, stroke (doubled), vascular disease, age 65–74 years, and sex category] was 1.54. Of the cohort, 22 (20.95%) were intolerant to Class I or Class III antiarrhythmic drug therapy, while the remaining patients had experienced failure of AAD therapy.

**Table 3:** Baseline patients characteristics.

Characteristics	Value (*n* = 105)
Age, years	63.70 ± 8.33
Male, *n*	63
BMI, mean ± SD	25.76 ± 3.96
Smoking and alcohol history, *n*	41
Medical history, *n* (%)	
CAD (MI/CABG)	4 (3.81%)
Diabetes	23 (21.90%)
Hypertension	71 (67.62%)
Dyslipidemia	11 (10.48%)
Sleep apnea	1 (0.95%)
Stroke or TIA	11 (10.48%)
COPD	1 (0.95%)
Echocardiographic parameters	
LA diameter, mm	33.90 ± 2.60
Left ventricular ejection fraction, %	61.34 ± 5.15
Preprocedural antiarrhythmic medication use, *n* (%)	
Class I	7 (6.67%)
Class III	15 (14.28%)
CHA₂DS₂-VASc score	1.54
HAS-BLED score	1.20 ± 0.90

Values are number (percent) or mean ± SD. AF, atrial fibrillation; BMI, body mass index; CAD, coronary artery disease; MI, myocardial infarction; CABG, coronary artery bypass grafting; TIA, transient ischemic attack; COPD, chronic obstructive pulmonary disease; LA, left atrium.

### Procedural metrics

As shown in Table 4, 79 patients were treated with conscious sedation, while the remaining 26 patients received general anesthesia during the procedure. The mean total procedure time was 101.53 ± 37.70 min. In the subgroup analysis according to post-ablation voltage mapping status, the procedure time was 110.2 ± 32.0 min in patients who underwent post-ablation voltage mapping (*n* = 52) and 93.0 ± 42.0 min in those without post-ablation voltage mapping (*n* = 53). The ablation time (from the first PFA application to the last application) was 218.9 ± 1.9 s. The PV potentials were shown immediately before and after a PFA application, demonstrating acute pulmonary vein isolation (Figure 5).

**Table 4:** Procedural characteristics (*N* = 105).

Characteristics	Value
General anaesthesia, *n*	26
Conscious sedation, *n*	79
Ablation time, s (mean ± SD)	218.90 ± 1.90
Total procedure time, min (mean ± SD)	101.53 ± 37.70
With post-ablation voltage mapping, min (mean ± SD)	110.20 ± 32.00 (*n* = 52)
Without post-ablation voltage mapping, min (mean ± SD)	93.00 ± 42.00 (*n* = 53)

Values are *n* or mean ± SD. Total procedure time was defined as the interval from the first venous puncture to removal of the groin sheaths. Ablation time was defined as time of first PFA therapy application to last PFA therapy application.

**Figure 5:**
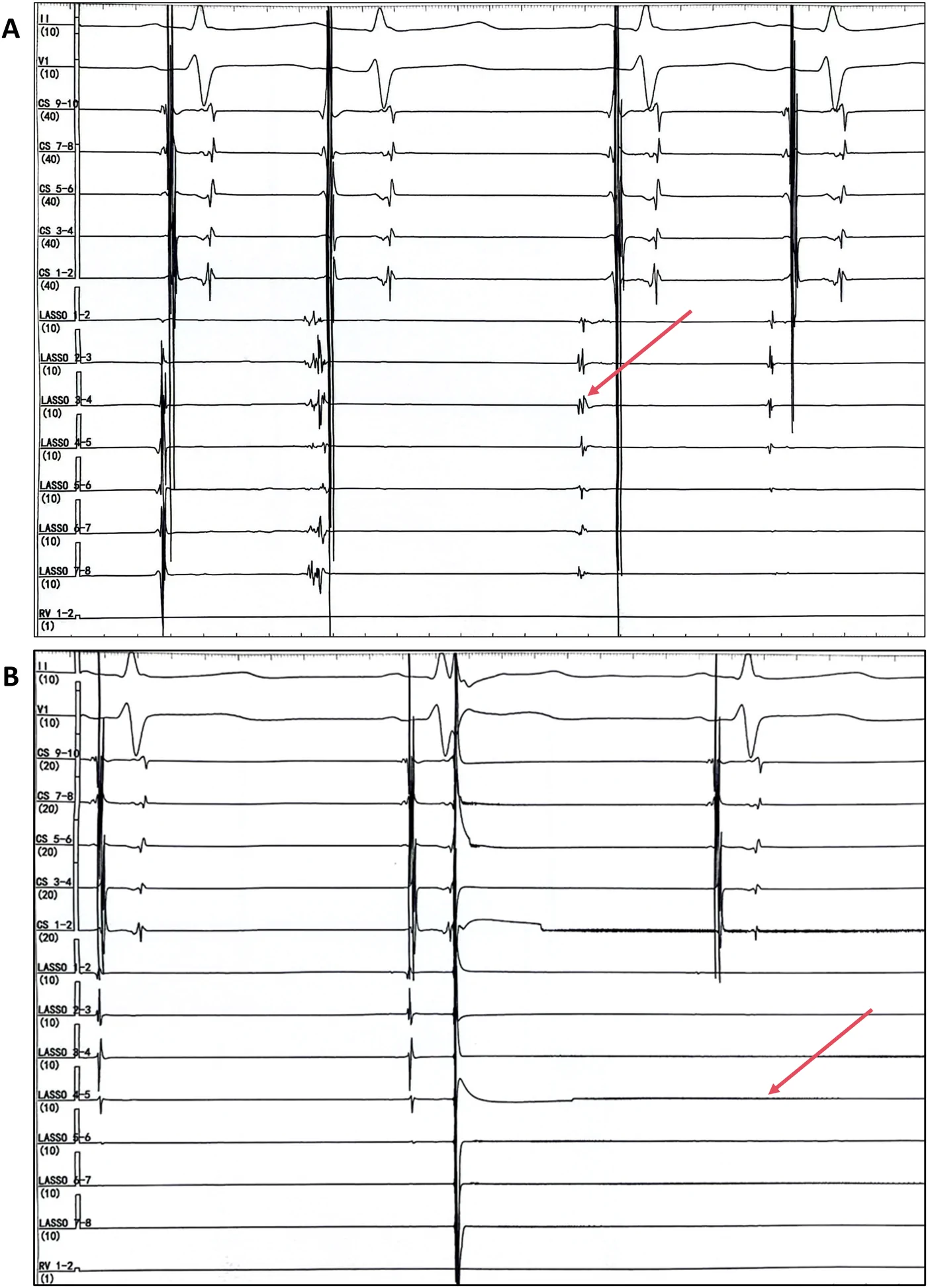
Electrocardiogram before and after pulsed field ablation. **(A)** Pulmonary vein potentials were clearly observed before ablation (red arrow). **(B)** Pulmonary vein potentials were completely eliminated after ablation, confirming successful acute pulmonary vein isolation (red arrow).

### Effectiveness endpoint

The primary effectiveness endpoint was 69.5% (95% CI, 60.1–77.5%) (the 1-year Kaplan–Meier estimate for freedom from AF/AFL/AT after a single procedure) (Figure 6). A detailed examination of atrial arrhythmia recurrence revealed that of the 32 patients with treatment failure, 11 had AF, 4 had AFL, and 17 had AT.

**Figure 6:**
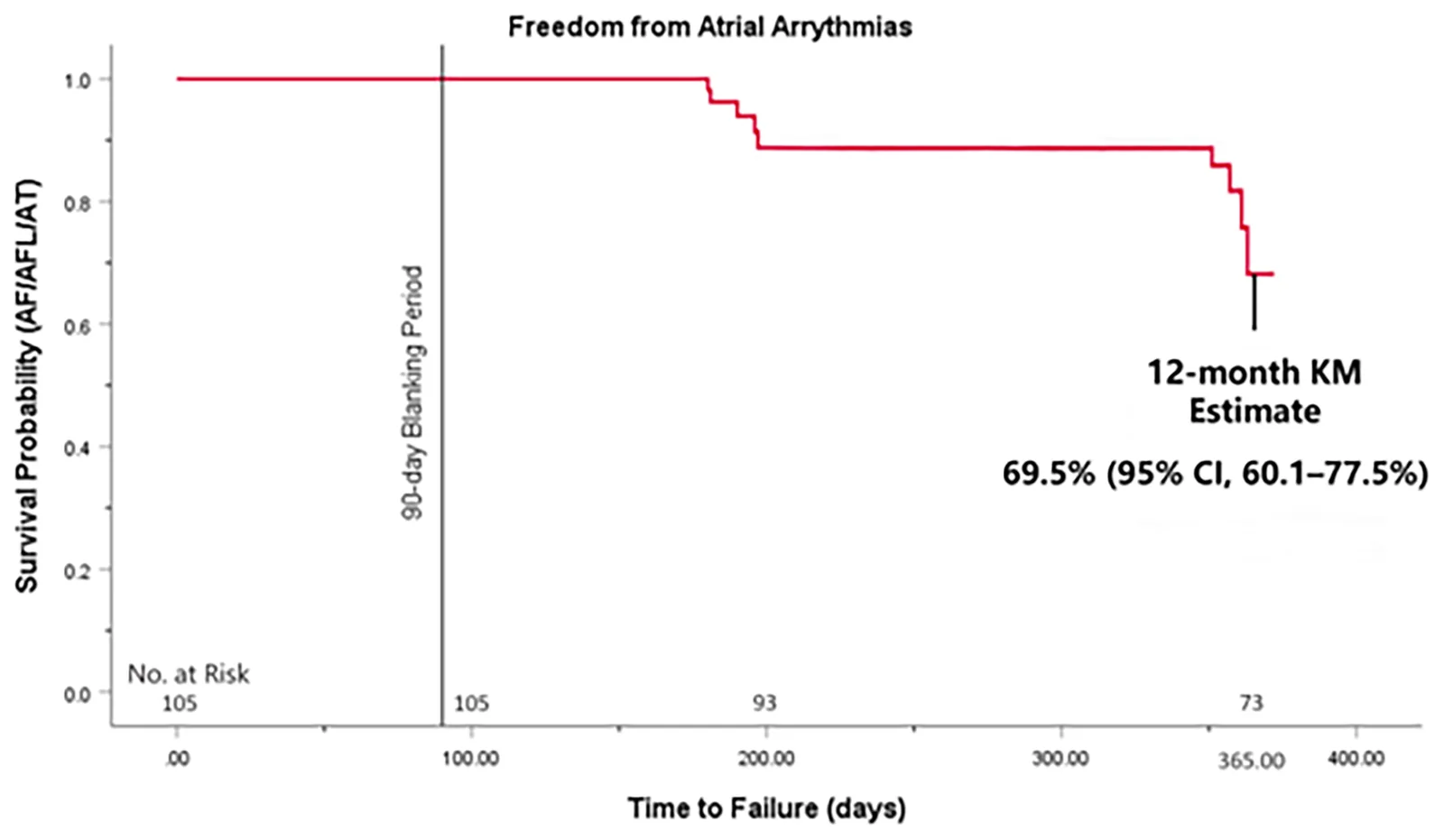
Freedom from atrial arrhythmias for the paroxysmal AF cohort. The Kaplan–Meier survival curves for freedom from atrial fibrillation (AF/AFL/AT) are shown for the paroxysmal AF cohort of 105 patients. KM indicates Kaplan–Meier.

A total of 420 PVs were ablated using the PFA catheter, and all 420 PVs (100%) were successfully acutely isolated. There were no instances of PV reconnection during the mandatory 20-minute waiting period. Post-ablation voltage mapping was performed in 52 of the 105 patients (49.5%) at the treating physician's discretion. Complete PV isolation was demonstrated in all PVs in these 52 patients, with no evidence of unintentional lesion formation. Post-ablation voltage maps revealed the level of electrical isolation to be at the PV antra (Figure 7).

**Figure 7:**
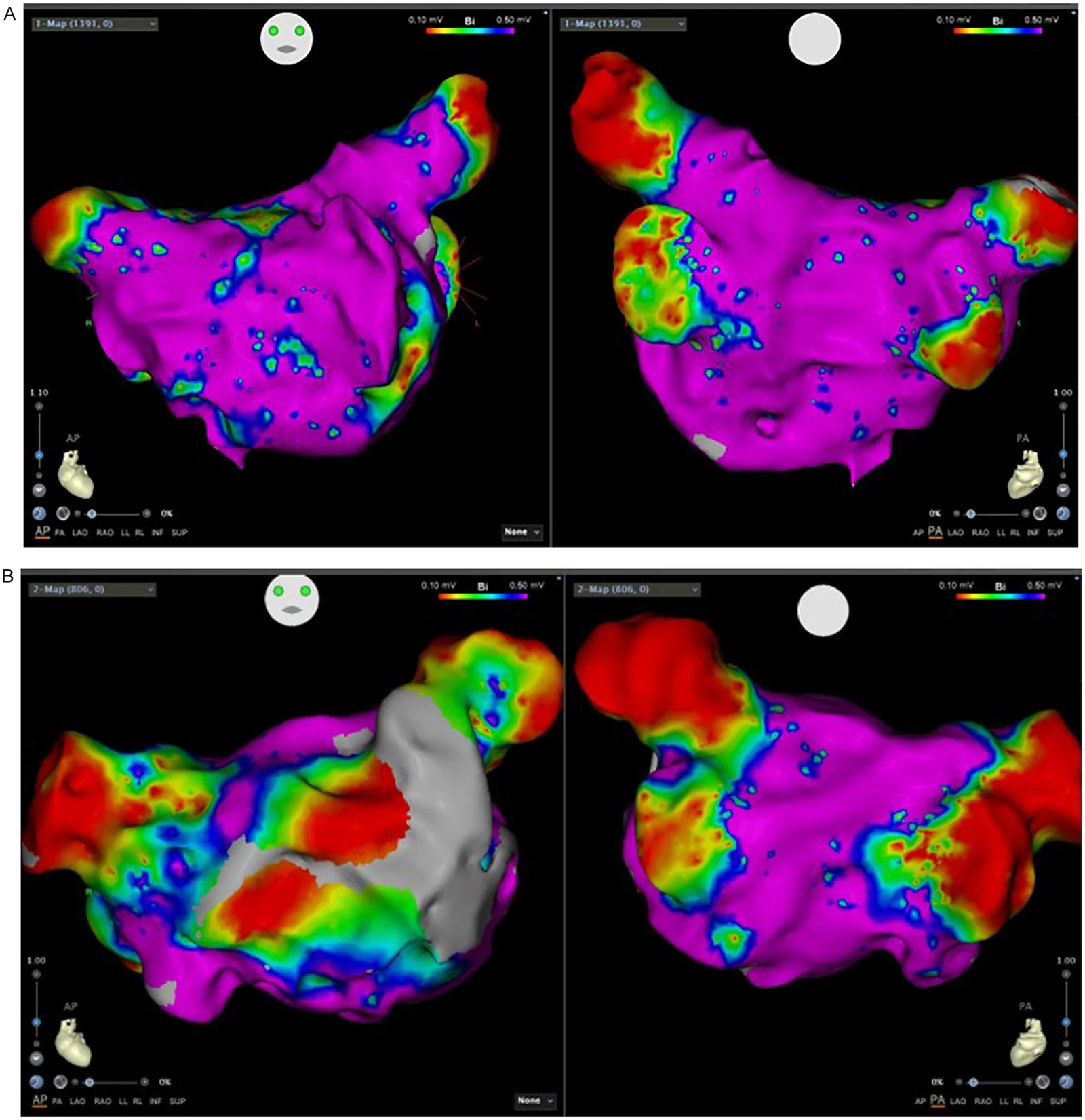
Three-dimensional electroanatomic maps illustrating voltage changes in pulmonary vein location potentials. **(A)** Mapping before ablation. **(B)** Mapping after ablation (after a 20-min waiting period) (red indicates low voltage, <0.1 mV; purple indicates preserved voltage, >0.5 mV).

### Safety endpoint

The primary safety endpoint occurred in one patient (0.95%, 1/105) who experienced a major procedure-related acute adverse event of coronary vasospasm. The vasospasm resolved completely following intracoronary vasodilator administration, resulting in no subsequent myocardial infarction or long-term sequelae.

During the 12-month follow-up, all patients underwent repeat CT or MRI at 3 and 6 months, and no cases of pulmonary vein stenosis or atrioesophageal fistula were reported. No patients exhibited evidence of esophageal injury on postoperative EGD. Details are provided in Table 5.

**Table 5:** Summary of serious adverse events (*N* = 105).

Serious Adverse Events	*n* (%)
Death	0 (0.00%)
Stroke	0 (0.00%)
Myocardial infarction	0 (0.00%)
Peripheral/ organ thromboembolism	0 (0.00%)
Cardiac tamponade/ perforation	0 (0.00%)
Coronary vasospasm	1 (0.95%)
Pericarditis	0 (0.00%)
Transient ischemic attack	0 (0.00%)
Persistent phrenic nerve palsy	0 (0.00%)
Pulmonary edema	0 (0.00%)
Pulmonary vein stenosis	0 (0.00%)
Atrioesophageal fistula	0 (0.00%)

Values are *n* (%); *N* indicates the total number of patients.

### Hemolysis and SCLs

Using the prespecified definition, hemolysis was observed in 92 of 105 patients (87.6%). Hp levels decreased from baseline to postoperative day 1 (124.6 ± 5.4 mg/dL vs. 112.8 ± 4.1 mg/dL, *P* < 0.0083). LDH levels increased from baseline to postoperative day 1 (256.4 ± 52.7 U/L vs. 398.2 ± 56.7 U/L, *P* < 0.0083). fHb also increased from baseline to immediately after the procedure (35.2 ± 8.5 mg/dL vs. 58.6 ± 12.3 mg/dL, *P* < 0.0083). Total bilirubin levels increased from baseline to postoperative day 1 (13.84 ± 6.89 μmol/L vs. 15.68 ± 7.42 μmol/L, P＞0.0083). In contrast, hemoglobin levels did not change from baseline to postoperative day 1 (141.2 ± 10.5 g/L vs. 138.6 ± 12.1 g/L, P＞0.0083), and creatinine levels did not change significantly from baseline to postoperative day 1 (70.88 ± 17.20 μmol/L vs. 70.82 ± 15.39 μmol/L, P＞0.0083). See Details are shown in Figure 8. No acute kidney injury (AKI) occurred. Notably, six patients (5.7%) exhibited a marked post-ablation increase in total bilirubin (25.41 ± 11.78 μmol/L), which was higher than that in the remaining 99 patients (15.09 ± 5.94 μmol/L). In these six patients, creatinine increased from 52.83 ± 4.62 to 70.17 ± 11.97 μmol/L; however, none met the Kidney Disease Improving Global Outcomes (KDIGO) criteria for AKI.

**Figure 8:**
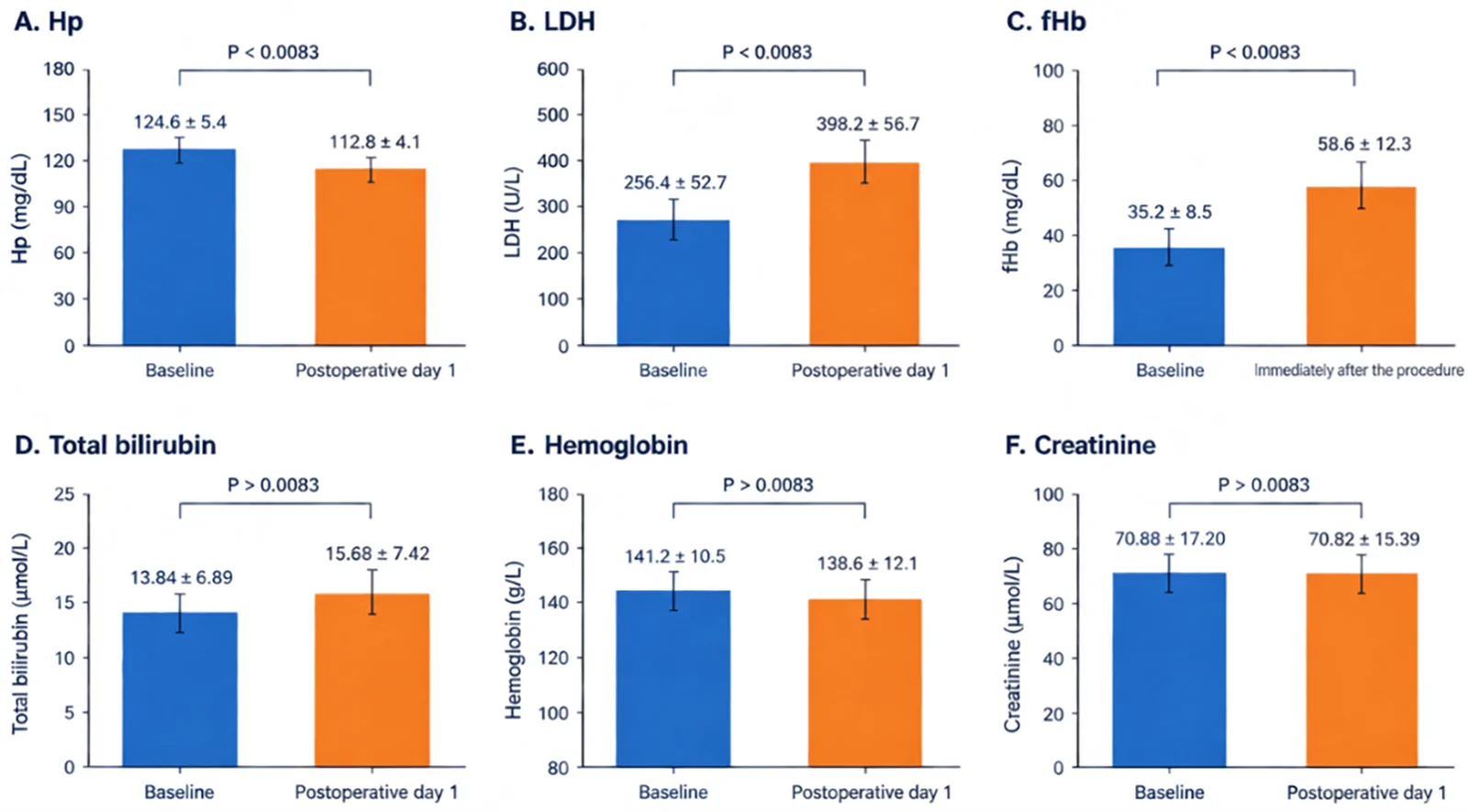
Hemolysis-related laboratory parameters at baseline and after the procedure. **(A)** Hp levels decreased significantly from baseline to postoperative day 1. **(B)** LDH levels increased significantly from baseline to postoperative day 1. **(C)** fHb levels increased significantly from baseline to immediately after the procedure. **(D)** Total bilirubin levels did not change significantly from baseline to postoperative day 1. **(E)** Hemoglobin levels did not change significantly from baseline to postoperative day 1. **(F)** Creatinine levels did not change significantly from baseline to postoperative day 1. Data are presented as mean ± SD. For secondary laboratory comparisons, statistical significance was defined as *P* ≤ 0.0083 after Bonferroni correction. Hp, haptoglobin; LDH, lactate dehydrogenase; fHb, free plasma hemoglobin.

SCLs developed in two patients (1.9%). Representative MRI findings in patients with and without SCLs are shown in Figure 9. Complete disappearance of the lesions was observed in two patients at the 3- and 6-month follow-up, whereas the remaining patients exhibited no significant MRI changes during postoperative follow-up.

**Figure 9:**
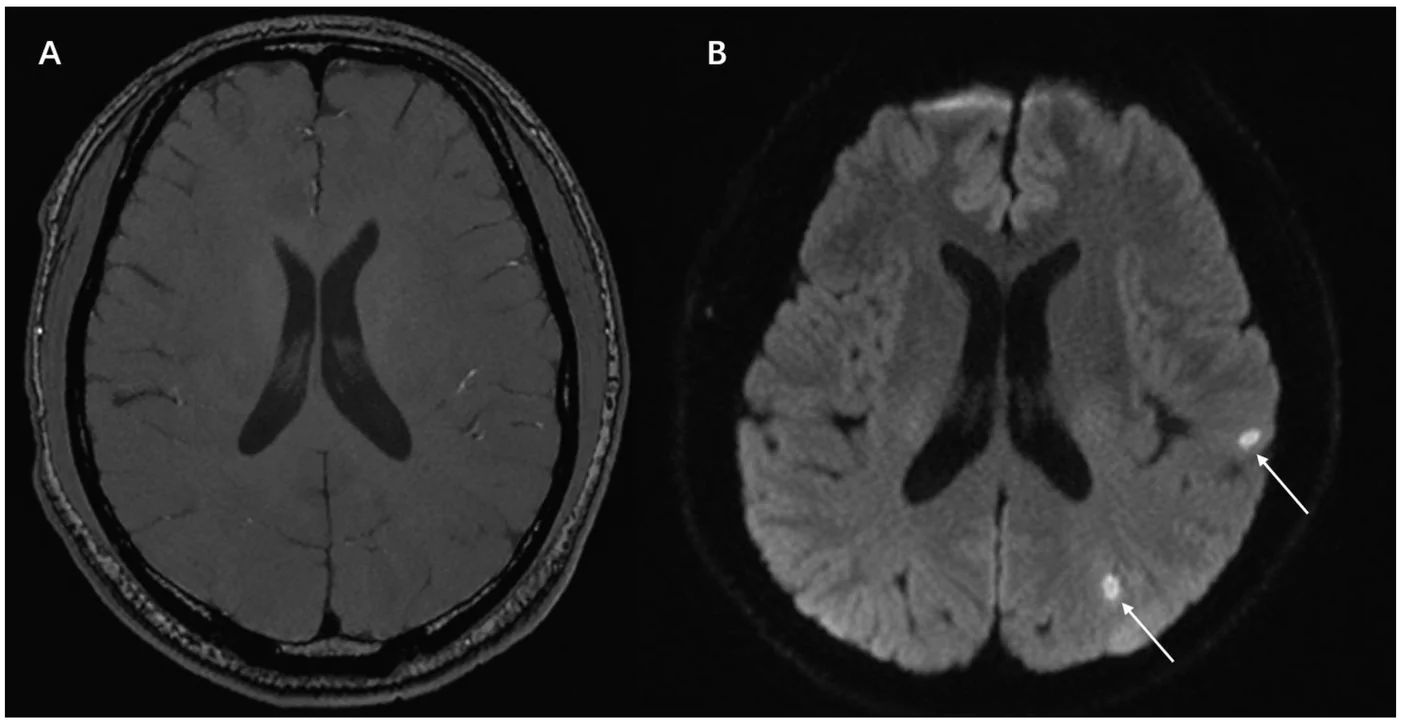
Representative brain MRI findings in patients with and without silent cerebral lesions (SCLs). **(A)** Representative brain MRI showing no evidence of SCL. **(B)** Postprocedural diffusion-weighted imaging (DWI) showing newly detected focal hyperintense lesions (arrows), consistent with SCLs.

## Discussion

In the clinical trial, we presented the efficacy and the safety of asymmetric biphasic PFA in patients with AF in the multicenter, prospective, single-arm trial. In 105 patients, 100% of PVs were acutely isolated and the incidence of the serious adverse events was low (0.95%). The primary effectiveness rate was 69.5% (95% CI, 60.1–77.5%). This study has demonstrated a very favorable procedural safety profile with a device- and procedure-related serious adverse event (SAE) rate of 0.95%. The study complied with follow-up and rigorous arrhythmia monitoring.

### Effectiveness and safety of asymmetric biphasic PFA

#### Effectiveness

In the clinical trial, we evaluated the effectiveness of asymmetric biphasic PFA for the electrical isolation of PVs in patients with paroxysmal AF. Even in the hands of multiple operators from multiple centers, acute PVI was achieved in all patients. Most importantly, the primary effectiveness outcome of freedom from AF/AFL/AT recurrence post-blanking was 69.5% (95% CI, 60.1–77.5%) after a single ablation procedure at 12-month follow-up. The 12-month effectiveness was within the range reported in previous studies of PFA technologies.

In the SINGLE SHOT CHAMPION Trial (19), patients with paroxysmal AF treated with PFA achieved a 1-year freedom from AF/AFL/AT recurrence rate of 62.9%. In the VOLT CE Mark Study (20), patients with paroxysmal AF achieved an 86.2% 6-month primary effectiveness endpoint success rate, and one repeat ablation procedure during the 90-day blanking period was permitted without being considered an endpoint failure. In the VOLT-AF IDE study (21), patients with paroxysmal AF undergoing initial PFA achieved a 12-month Kaplan–Meier estimated effectiveness rate of 81.1%, and one repeat ablation procedure during the 90-day blanking period was permitted by the protocol without affecting the primary effectiveness endpoint. In the PULSED AF Pivotal Trial (18), patients with paroxysmal AF achieved a 12-month Kaplan–Meier estimated effectiveness rate of 66.2%, and repeat pulsed field ablation during the 90-day blanking period was permitted without penalty to the primary effectiveness endpoint. The allowance of repeat ablation during the blanking period may have influenced the reported effectiveness rates in these studies, as early recurrences could be managed with additional ablation without being considered endpoint failures. In contrast to these studies, no patient underwent repeat ablation within the 90-day blanking period in our trial. It should be noted that these studies differed from the present study in terms of patient population and treatment setting, particularly with respect to persistent AF and repeat ablation; therefore, direct cross-study comparisons should be interpreted with caution.

#### Safety

There were no clinical manifestations of PFA-related esophageal injury, atrioesophageal fistula, phrenic nerve injury and PV stenosis. The result is consistent with our previous preclinical and clinical studies (15, 22). Only 1 patient developed coronary vasospasm with associated ST-segment elevation on postoperative day 3. The patient had experienced several similar episodes before enrollment, and coronary angiography revealed a pre-existing high-grade atherosclerotic coronary artery lesion. Although the underlying coronary atherosclerotic lesion may have contributed to the occurrence of vasospasm, a potential contributory role of the PFA procedure cannot be completely excluded. Indeed, there have been published case reports of ST-segment elevation after PFA (6, 23). Nitroglycerin appears effective for both the treatment and prevention of PFA-related coronary spasm. Reddy et al. found that severe spasm occurred in all patients undergoing cavotricuspid isthmus (CTI) ablation without prophylaxis and was relieved by intracoronary nitroglycerin (1–2 mg), whereas prophylactic intracoronary or intravenous nitroglycerin prevented severe spasm (24). Similarly, Malyshev et al. showed that a 3-mg bolus followed by 2 mg every 2 min reduced the incidence of severe spasm from 80% to 0% and prevented ST-segment elevation (25). Therefore, ST-segment elevation during or after PFA should prompt urgent coronary angiography and intracoronary nitroglycerin administration. For planned ablation near coronary arteries, such as the CTI or mitral isthmus, prophylactic parenteral nitroglycerin may also be considered, with phenylephrine administered when necessary to mitigate nitrate-induced hypotension.

#### SCLs-related complications

Gas bubble generation during PFA for cardiac procedures has emerged as an important safety consideration, primarily because these gaseous microbubbles may serve as microembolic sources and are therefore implicated in SCLs and, in rare cases, stroke (26–29). In these days, multiple clinical investigations have already reported some SCLs and cerebrovascular events following PFA-based ablation. In the neurological assessment subgroup of the ADVENT trial (30), 3 of 34 patients treated with PFA were confirmed by the core laboratory to have silent cerebral embolism (SCE)/SCL. In a prospective randomized controlled trial (31), 4 of 31 patients treated with PFA developed SCL, yielding an incidence of 12.90%. In the trial by Sánchez-Gómez et al. (32), 3 of 45 patients treated with PFA developed new brain lesions (2 SCLs and 1 TIA), yielding an incidence of 6.7%.

These procedure-related cerebral events should be interpreted within the broader context of ablation's overall stroke-risk profile. A recent large meta-analysis pooling 18 randomized controlled trials (*n* = 5,877) demonstrated that catheter ablation significantly reduces long-term stroke risk compared with medical therapy (RR 0.63, 95% CI 0.45–0.87) (33). However, a separate systematic review and meta-analysis of catheter and surgical ablation reported a markedly elevated risk of ischemic stroke occurring within 30 days of the procedure (RR 6.81, 95% CI 1.56–29.8), which substantially attenuated the net stroke benefit when periprocedural events were included (RR for any ischemic stroke 0.77, 95% CI 0.55–1.09) (34). Taken together, these findings indicate that there is a clinically relevant vulnerability to thromboembolic complications during the periprocedural period, despite the potential long-term reduction in stroke risk following AF ablation. Against this background, technical strategies aimed at minimizing procedure-related embolic sources, such as the waveform and electrode optimizations described below, warrant particular attention in PFA.

No bubbles were observed on intracardiac echocardiography during the procedure, and the incidence of SCLs was low, which may be related to the improvements implemented in our PFA system. Firstly, optimize pulse waveforms. Based on our previous bench tests, biphasic waveforms tend to produce fewer microbubbles than monophasic configurations, and asymmetric biphasic waveform has better ablation depth than biphasic symmetric waveforms. So asymmetric biphasic short-waveforms are kept in our PFA system. Secondly, electrode design is designed avoiding sharp geometries and regions prone to excessive current concentration, which can otherwise promote localized arcing and bubble generation.

#### Hemolysis-related complications

Intravascular hemolysis is an established, energy-driven side effect of PFA in electrophysiology, brought to light following reports of AKI in two patients undergoing arrhythmia treatment (35). Clinical data correlates this red blood cell destruction with secondary conditions like hemoglobinuria and, less frequently, severe renal impairment (36, 37). Hemolysis occurred frequently after PFA. A retrospective cohort study demonstrated substantial differences in the severity of hemolysis among catheter platforms. The incidence of hemolysis was 88% with the Sphere−9 catheter, compared with 97% for the Farawave and Varipulse catheters and 100% for the PulseSelect catheter (16). The lower hemolysis in our cohort appear to result from the following modifications to our procedural approach: (1) Optimizing Energy Delivery: Utilizing asymmetric biphasic waveforms can curb erythrocyte electroporation without sacrificing the therapeutic success of the ablation (38). (2) Structural and Contact Countermeasures: Hemolysis occurs when electrical currents stray from myocardial tissue into the bloodstream, fracturing blood cells. Because blood features superior electrical conductivity compared to cardiac tissue, it preferentially absorbs PFA energy—a vulnerability exacerbated by direct electrode exposure to blood pool volumes. Employing basket catheter configurations alongside single-sided electrode configurations ensures robust tissue coupling, which dampens localized current densities and shields red blood cells from peak energy zones (16, 39). (3) Periprocedural Fluid Protocols: Administering strategic hydration throughout and following the operation effectively lowers the incidence of downstream hemoglobinuria and AKI (37).

## Conclusion

This multicenter prospective clinical trial demonstrated that the novel asymmetric biphasic PFA system achieved complete acute pulmonary vein isolation with favorable safety in patients with paroxysmal atrial fibrillation. At 12 months, the system showed encouraging effectiveness. Only one case of transient coronary vasospasm occurred, and no pulmonary vein stenosis, phrenic nerve injury, or atrioesophageal fistula was observed. SCLs occurred at a low frequency. Hemolysis was observed in some patients but did not result in AKI. These findings support the safety and feasibility of this novel PFA system.

### Limitations

First, this was a multicenter, prospective, single-arm study without a comparator group; therefore, the efficacy and safety findings should be interpreted within the context of the study design, and larger comparative multicenter studies are needed to further validate the generalizability of our results. Second, the lack of continuous monitoring with an implanted device may have limited the complete detection of recurrent atrial arrhythmias and, consequently, the assessment of changes in AF burden before and after ablation. Third, the sample size was relatively limited, especially for detecting rare adverse events; therefore, the absence of observed rare complications should not be interpreted as evidence that such events cannot occur. Fourth, patients with baseline eGFR <60 mL/min/1.73 m² were excluded from the study; consequently, the hemolysis and renal safety findings may not be generalizable to patients with pre-existing renal impairment. Finally, continued optimization of the asymmetric biphasic pulse parameters, particularly pulse duration, may further improve the clinical application of this technology

## Data Availability

The raw data supporting the conclusions of this article will be made available by the authors, without undue reservation.

## References

[1] Hunter DW Kostecki G Fish JM Jensen JA Tandri H. *In vitro* cell selectivity of reversible and irreversible: electroporation in cardiac tissue. Circ Arrhythm Electrophysiol. (2021) 14(4):e008817. 10.1161/CIRCEP.120.008817 33729827

[2] Pu S Liu F Chen Y Luo C Li P Chen Y. Single-shot' Pulmonary vein isolation using a novel lotos pulsed field ablation catheter: a pre-clinical evaluation of feasibility, safety, and 30-day efficacy. Europace. (2023) 26(1):euad362. 10.1093/europace/euad362 38109928 PMC10757452

[3] Yu F Dong X Ding L Reddy V Tang M. Pulsed field ablation for pulmonary vein isolation: preclinical safety and effectiveness of a novel hexaspline ablation catheter. J Cardiovasc Electrophysiol. (2023) 34(11):2195–202. 10.1111/jce.16067 37712346

[4] Cochet H Nakatani Y Sridi-Cheniti S Cheniti G Ramirez FD Nakashima T. Pulsed field ablation selectively spares the oesophagus during pulmonary vein isolation for atrial fibrillation. Europace. (2021) 23(9):1391–9. 10.1093/europace/euab090 33961027 PMC8427383

[5] Reddy VY Dukkipati SR Neuzil P Anic A Petru J Funasako M. Pulsed field ablation of paroxysmal atrial fibrillation: 1-year outcomes of IMPULSE, PEFCAT, and PEFCAT II. JACC Clin Electrophysiol. (2021) 7(5):614–27. 10.1016/j.jacep.2021.02.014 33933412

[6] Turagam MK Neuzil P Schmidt B Reichlin T Neven K Metzner A. Safety and effectiveness of pulsed field ablation to treat atrial fibrillation: one-year outcomes from the MANIFEST-PF registry. Circulation. (2023) 148(1):35–46. 10.1161/CIRCULATIONAHA.123.064959 37199171

[7] Ekanem E Reddy VY Schmidt B Reichlin T Neven K Metzner A. Multi-national survey on the methods, efficacy, and safety on the post-approval clinical use of pulsed field ablation (MANIFEST-PF). Europace. (2022) 24(8):1256–66. 10.1093/europace/euac050 35647644 PMC9435639

[8] García-Sánchez T Amorós-Figueras G Jorge E Campos MC Maor E Guerra JM. Parametric study of pulsed field ablation with biphasic waveforms in an *in vivo* heart model: the role of frequency. Circ Arrhythm Electrophysiol. (2022) 15(10):e010992. 10.1161/CIRCEP.122.010992 36178752

[9] Cvetkoska A Maček-Lebar A Polajžer T Reberšek M Upchurch W Iaizzo PA. The effects of interphase and interpulse delays and pulse widths on induced muscle contractions, pain and therapeutic efficacy in electroporation-based therapies. J Cardiovasc Dev Dis. (2023) 10(12):490. 10.3390/jcdd10120490 38132658 PMC10744272

[10] Chen S Narayan SM Boveda S Neven K Ruwald MH Martinek M. 2025 International expert practical guide on the use of the pentaspline pulsed field ablation system in atrial fibrillation ablation procedures. Circ Arrhythm Electrophysiol. (2025) 18(8):e013977. 10.1161/CIRCEP.125.013977 40740131 PMC12533784

[11] Pakhomov AG Grigoryev S Semenov I Casciola M Jiang C Xiao S. The second phase of bipolar, nanosecond-range electric pulses determines the electroporation efficiency. Bioelectrochemistry. (2018 Aug) 122:123–33. 10.1016/j.bioelechem.2018.03.014 PMC5984177 29627664

[12] Malyško-Ptašinskė V Nemeikaitė-Čėnienė A Radzevičiūtė-Valčiukė E Mickevičiūtė E Malakauskaitė P Lekešytė B. Threshold interphase delay for bipolar pulses to prevent cancellation phenomenon during electrochemotherapy. Int J Mol Sci. (2024) 25(16):8774. 10.3390/ijms25168774 PMC11354671 39201461

[13] Vázquez-Calvo S Poletaev V Verma A. Latest advances and ongoing challenges in pulsed field ablation. Arrhythm Electrophysiol Rev. (2026) 15:e06. 10.15420/aer.2025.32 PMC12983143 41835109

[14] Ye X Liu S Yin H He Q Xue Z Lu C. Study on optimal parameter and target for pulsed-field ablation of atrial fibrillation. Front Cardiovasc Med. (2021) 8:690092. 10.3389/fcvm.2021.690092 34621795 PMC8490619

[15] Bi S Jia F Lv C He Q Xu X Xue Z. Preclinical study of biphasic asymmetric pulsed field ablation. Front Cardiovasc Med. (2022) 9:859480. 10.3389/fcvm.2022.859480 35402543 PMC8987372

[16] Gianni C Al-Ahmad A Elchouemi M Fazia VML Mohanty S Allison JD. Pulsed field ablation-related hemolysis: comparison between technologies. Circ Arrhythm Electrophysiol. (2025) 18(12):e014021. 10.1161/CIRCEP.125.014021 41328578 PMC12711278

[17] Calkins H Hindricks G Cappato R Kim Y-H Saad EB Aguinaga L. 2017 HRS/EHRA/ECAS/APHRS/SOLAECE expert consensus statement on catheter and surgical ablation of atrial fibrillation: executive summary. J Interv Card Electrophysiol. (2017) 50(1):1–55. 10.1007/s10840-017-0277-z 28914401 PMC5633646

[18] Verma A Haines DE Boersma LV Sood N Natale A Marchlinski FE. Pulsed field ablation for the treatment of atrial fibrillation: PULSED AF pivotal trial. Circulation. (2023) 147(19):1422–32. 10.1161/CIRCULATIONAHA.123.063988 36877118 PMC10158608

[19] Reichlin T Kueffer T Badertscher P Jüni P Knecht S Thalmann G. Pulsed field or cryoballoon ablation for paroxysmal atrial fibrillation. N Engl J Med. (2025) 392(15):1497–507. 10.1056/NEJMoa2502280 40162734

[20] Tilz RR Chierchia GB Gunawardene M Sanders P Haqqani H Kalman J. Safety and effectiveness of the first balloon-in-basket pulsed field ablation system for the treatment of atrial fibrillation: VOLT CE mark study 6-month results. Europace. (2025) 27(4):euaf072. 10.1093/europace/euaf072 40163671 PMC12036658

[21] Verma A Lo M Woods CE Hussein AA Gambhir A Sundaram S. Balloon-in-basket pulsed field ablation for pulmonary vein isolation: 1-year outcomes of the VOLT-AF IDE study. JACC Clin Electrophysiol. (2026) 12(6):1287–99. 10.1016/j.jacep.2026.01.006 41649432

[22] Chen B Lv C Cui Y Lu C Cai H Xue Z. A pilot clinical assessment of biphasic asymmetric pulsed field ablation catheter for pulmonary vein isolation. Front Cardiovasc Med. (2024) 11:1266195. 10.3389/fcvm.2024.1266195 38385135 PMC10879394

[23] Reddy VY Peichl P Anter E Rackauskas G Petru J Funasako M. A focal ablation catheter toggling between radiofrequency and pulsed field energy to treat atrial fibrillation. JACC Clin Electrophysiol. (2023) 9(8 Pt 3):1786–801. 10.1016/j.jacep.2023.04.002 37227340

[24] Reddy VY Petru J Funasako M Kopriva K Hala P Chovanec M. Coronary arterial spasm during pulsed field ablation to treat atrial fibrillation. Circulation. (2022) 146(24):1808–19. 10.1161/CIRCULATIONAHA.122.061497 36134574

[25] Malyshev Y Neuzil P Petru J Funasako M Hala P Kopriva K. Nitroglycerin to ameliorate coronary artery spasm during focal pulsed-field ablation for atrial fibrillation. JACC Clin Electrophysiol. (2024) 10(5):885–96. 10.1016/j.jacep.2023.12.015 38385916

[26] Verma A Asivatham SJ Deneke T Castellvi Q Neal RE 2nd. Primer on pulsed electrical field ablation: understanding the benefits and limitations. Circ Arrhythm Electrophysiol. (2021) 14(9):e010086. 10.1161/CIRCEP.121.010086 34538095

[27] McBride S Avazzadeh S Wheatley AM O'Brien B Coffey K Elahi A. Ablation modalities for therapeutic intervention in arrhythmia-related cardiovascular disease: focus on electroporation. J Clin Med. (2021) 10(12):2657. 10.3390/jcm10122657 34208708 PMC8235263

[28] Van Es R Groen MHA Stehouwer M Doevendans PA Wittkampf FHM Neven K. *In vitro* analysis of the origin and characteristics of gaseous microemboli during catheter electroporation ablation. J Cardiovasc Electrophysiol. (2019) 30(10):2071–9. 10.1111/jce.14091 31347222

[29] Ranjbartehrani P. Microbubble formation and hemolysis in pulsed field ablation for treating arrhythmia: risks, mechanisms, and prevention. Pacing Clin Electrophysiol. (2026) 49(1):52–7. 10.1111/pace.70035 41347755 PMC12813644

[30] Patel C Gerstenfeld EP Gupta SK Winterfield J Woods C Natale A. Comparison of cerebral safety after atrial fibrillation using pulsed field and thermal ablation: results of the neurological assessment subgroup in the ADVENT trial. Heart Rhythm. (2024) 21(11):2103–9. 10.1016/j.hrthm.2024.05.048 38823667

[31] Ding P Chen W Shen J Qiu B Hu X Xie L. Incidence of silent cerebral lesions during pulsed field ablation for paroxysmal atrial fibrillation. Ann Med. (2026) 58(1):2634472. 10.1080/07853890.2026.2634472 41774050 PMC12958377

[32] Sánchez-Gómez JM Ortiz-Fernández Á Soler-Martínez J Gabaldón-Pérez A Iraola-Viana D Martínez-Brotons Á. Incidence of brain lesions following pulsed field ablation for atrial fibrillation. Heart Rhythm. (2026) 23(1):8–15. 10.1016/j.hrthm.2025.02.020 40736426

[33] Providencia R Ali H Barra S Creta A Kukendrarajah K Kanagaratnam P. Ablation of atrial fibrillation and risk of stroke: a meta-analysis. Heart Rhythm. (2026) 23(1):44–55. 10.1016/j.hrthm.2025.05.021 40398548

[34] Montané B Zhang S Wolfe JD Prime S Luo C Cooper DH. Catheter and surgical ablation for atrial fibrillation: a systematic review and meta-analysis. Ann Intern Med. (2025) 178(8):1138–49. 10.7326/ANNALS-25-00253 40587868

[35] Yuan J Wang Z Yu Y Peng H Shi S He F. The protective impact of Gd-DTPA on hemolysis in pulsed field ablation for atrial fibrillation. JACC Clin Electrophysiol. (2024) 10(12):2741–3. 10.1016/j.jacep.2024.09.028 39570267

[36] Auf der Heiden C Bejinariu AG Kelm M Spieker M Rana O. Hemolysis after pulsed-field ablation in pulmonary vein isolation for atrial fibrillation: a prospective controlled trial. Heart Rhythm. (2026) 23(2):e209–16. 10.1016/j.hrthm.2025.04.038 40280310

[37] He J Li K He C Zhang L Yang D Qin H. Evaluation of dose and contact of a circular pulsed field ablation catheter in a porcine model: impact on hemolysis and renal function. Heart Rhythm. (2026) 23(2):405–17. 10.1016/j.hrthm.2025.07.042 40744191

[38] Kuraoka S Nozoe M Mannoji H Miyake R Uchikawa T Ishikita A. Differential subclinical hemolysis after pulsed field ablation using the FARAPULSE pentaspline catheter vs the PulseSelect circular multi-electrode array catheter. Heart Rhythm O2. (2025) 6(12):1911–8. 10.1016/j.hroo.2025.08.042 41541714 PMC12800815

[39] Yoshimura S Kaseno K Kodama A Nishiuchi S Hattori K Masuyama T. Effect of pulsed field ablation delivered from noncontact catheter electrodes on hemolysis: a tissue proximity indication-based analysis. Heart Rhythm O2. (2025) 6(11):1716–21. 10.1016/j.hroo.2025.09.002 41357276 PMC12675092

